# Revisited analysis of a SHIVA01 trial cohort using functional mutational analyses successfully predicted treatment outcome

**DOI:** 10.1002/1878-0261.12180

**Published:** 2018-03-30

**Authors:** Maud Kamal, Gabi Tarcic, Sylvain Dureau, Oded Edelheit, Zohar Barbash, Charlotte Lecerf, Claire Morel, Benjamin Miron, Celine Callens, Nicolas Servant, Ivan Bieche, Michael Vidne, Christophe Le Tourneau

**Affiliations:** ^1^ Department of Drug Development and Innovation Institut Curie Paris & Saint‐Cloud France; ^2^ NovellusDx Jerusalem Israel; ^3^ Department of Biostatistics Institut Curie Paris France; ^4^ Department of genetics Institut Curie Paris France; ^5^ Institut Curie/INSERM U900 Saint‐Cloud France; ^6^ Versailles‐Saint‐Quentin‐en‐Yvelines University Montigny‐le‐Bretonneux France

**Keywords:** drug response, *in vitro* functional assay, mutation, oncogenic activity, variant with unknown significance

## Abstract

It still remains to be demonstrated that using molecular profiling to guide therapy improves patient outcome in oncology. Classification of somatic variants is not straightforward, rendering treatment decisions based on variants with unknown significance (VUS) hard to implement. The oncogenic activity of VUS and mutations identified in 12 patients treated with molecularly targeted agents (MTAs) in the frame of SHIVA01 trial was assessed using Functional Annotation for Cancer Treatment (FACT). MTA response prediction was measured *in vitro*, blinded to the actual clinical trial results, and survival predictions according to FACT were correlated with the actual PFS of SHIVA01 patients. Patients with positive prediction had a median PFS of 5.8 months versus 1.7 months in patients with negative prediction (*P* < 0.05). Our results highlight the role of the functional interpretation of molecular profiles to predict MTA response.

**Abbreviations**
FACT: Functional Annotation for Cancer Treatment
MT: mutation
MTA: molecularly targeted agent
NCR: nuclear‐to‐cytoplasmic ratio
PFS: progression‐free survival
SNV: single‐nucleotide variant
VUS: variant with unknown significance
WT: wild‐type

## 1 Introduction

The use of molecularly targeted agents (MTAs) in oncology has shown improvement of patients' survival in different cancer types when the corresponding molecular alteration is present (Hyman *et al*., 2017). It remains to be demonstrated whether using precision medicine technologies to guide therapy improves patient outcome (Le Tourneau *et al*., 2014).

Results of retrospective analyses of tumor molecular screening programs (Tsimberidou *et al*., 2014) and nonrandomized clinical trials (Massard *et al*., 2017; Von Hoff *et al*., 2010) assessing the clinical utility of using high‐throughput technologies were not confirmed in the SHIVA01 randomized trial (Le Tourneau *et al*., 2015). The SHIVA01 trial was the first prospective, randomized precision medicine trial comparing targeted therapy based on tumor molecular profile versus treatment by physician's choice in patients with diverse types of metastatic cancer that had failed standard‐of‐care treatment. In SHIVA01, eleven MTAs were given depending on molecular analyses performed on an on‐purpose tumor biopsy of a metastatic site. The SHIVA01 trial was negative for its primary endpoint (i.e., progression‐free survival [PFS]), where no statistically significant difference in PFS was reported between the MTA and control arms (Le Tourneau *et al*., 2015). Encouraging results were however inferred from SHIVA01 subgroup analyses where 1) patients whose tumors harbored a molecular alteration involving the receptor tyrosine kinase/mitogen‐activated protein kinases (RTK/MAPK) pathway had an improved PFS (Le Tourneau *et al*., 2014, 2015), and 2) patients who crossed over in SHIVA01 compared favorably to results (Belin *et al*., 2017) obtained in the von Hoff study and in MOSCATO with PFS on matched targeted therapy to PFS on last nonmatched treatment ratio exceeding 1.3 in 37% of patients (Massard *et al*., 2017; Von Hoff *et al*., 2010).

One of the main concerns related to the negative results of SHIVA01 is the need of a refined treatment algorithm taking into account multiple tumor's alterations of each patient and the driver molecular events that prove to be relevant for each MTA. Classification of somatic variants is not straightforward, rendering treatment algorithms hard to implement. A four‐tiered system categorizes variants depending on the their clinical significance (Li *et al*., 2017). Many variants have unknown significance (VUS) and usually require the use of various *in silico* prediction algorithms, rarely in agreement, to predict whether an alteration in a gene will change the structure and function of the altered protein (Adzhubei *et al*., 2013; Sim *et al*., 2012). The addition of functional information on identified molecular alterations, or combinations of alterations, and their response to MTAs may therefore improve treatment outcomes.

## 2 Methods

### 2.1 Patients

The twelve SHIVA01 patients selected for the FACT analyses are patients whose tumors harbored a molecular alteration involving the receptor tyrosine kinase/mitogen‐activated protein kinases (RTK/MAPK) pathway.

Patients enrolled in the SHIVA01 trial were patients older than 18 years with any kind of recurrent or metastatic solid tumor for whom standard‐of‐care therapy had failed, provided their disease was accessible for a biopsy or resection of a metastatic site (Le Tourneau *et al*., 2015).

All patients provided written informed consent. The study was approved by the Ile‐de‐France ethics committee. The trial was carried out in accordance with the Declaration of Helsinki, the Good Clinical Practice guidelines of the International Conference on Harmonization, and relevant French and European laws and directives. Patients were eligible for randomization if one or several molecular alterations were identified that matched one of the available MTA regimens.

The twelve SHIVA01 patients selected for the FACT analyses were patients whose tumors harbored a molecular alteration involving the receptor tyrosine kinase/mitogen‐activated protein kinases (RTK/MAPK) pathway. In total, 20 SHIVA01 patients were treated following randomization or crossover with MTAs in the RTK/MAPK pathway, and 12 patients harbored VUS or known mutations in this signaling pathway and were suitable for FACT analyses.

### 2.2 Patients' molecular analyses

Molecular profiles for patient tumors based on samples from a mandatory biopsy or resection of a metastasis included assessment of variants by targeted next‐generation sequencing (AmpliSeq Cancer Panel on an Ion Torrent/Personal Genome Machine System; Life Technologies, Carlsbad, CA, USA).

### 2.3 Variant analyses

For mutation analyses, sequencing reads were aligned on the human reference genome (hg19) using the Torrent Mapping Alignment Program software (Life Technologies^®^). The variants were detected using the Variant Caller software (Life Technologies^®^) and annotated using the ANNOVAR pipeline. The variants were filtered according to their frequency (> 4% for single‐nucleotide variant (SNV) and > 10% for indels), strand ratio (> 0.2), and reads coverage (> 30 × for SNV and 100 × for indels). Variants of unknown significance were variants lacking *in silico* analyses and with no reports available in the literature.

### 2.4 NovellusDx Functional Annotation for Cancer Treatment (FACT)

Patient variants were functionally characterized using an *in vitro* cell‐based assay (FACT) designed to analyze oncogenic activity based on activation of signaling pathways (oncogenic activity prediction) of variants and subsequent inhibition via MTA treatment (MTA response prediction) at a Clinical Laboratory Improvement Amendments‐certified laboratory (NovellusDx, Jerusalem, Israel). Patient variants were generated on a wild‐type expression vector backbone. Then, the variants and a specific signaling pathway reporter were transfected into a live‐cell assay. The signaling pathway reporter here was a fluorescent‐tagged signaling protein which translocates from the cytoplasm to the nucleus upon pathway activation. The live‐cell assay was then scanned by a fluorescent microscope to detect reporter localization.

Variant synthesis was performed using the Q5 site‐directed mutagenesis kit (New England Biolabs, Cat. #E0554S) and verified using Sanger sequencing. HeLa cells were seeded in 96‐well poly‐L‐lysine‐coated, transparent‐bottom plate. Twenty‐four hours after seeding, cells were transfected with a mixture of plasmids (wild‐type (WT), known mutation, or patient mutations of the relevant gene) and an EGFP‐tagged reporter in six repeats using the FuGENE HD reagent (Promega, Cat. #E2312). After transfection, cells were incubated for 24 h to allow adequate expression of the gene constructs. The plates were then fixated using paraformaldehyde, and a nuclear stain (DAPI) was performed. Plates were imaged using a NIKON Ti Eclipse microscope and NIS‐Elements software. The images are processed by an integrated image analysis software system for high‐throughput segmentation of cells and corresponding nuclei that defines cell borders and nucleus borders and quantifies the fluorescence intensity of the reporter in each one of these compartments. The system is composed of robust image enhancement, followed by multispectral identification of putative cells using a Gaussian mixture model, followed by cross‐spectral watershed that effectively segments clustered cells, and a role‐based refinement using statistical morphological attributes of the cells. The output of this process is a calculated nuclear‐to‐cytoplasmic ratio (NCR) of each reporter per cell analyzed. The median NCR of all the transfected cells in a well is taken to be the NCR of the condition in the well. Each condition is repeated in six wells, and using the median NCR of each well, we obtain the average NCR of the condition. Each gene was tested for two canonical pathways that are known to be activated and that are successfully measured in the FACT platform.

Signaling pathway activation was measured using fluorescently tagged proteins that are part of the pathway and which shuttle from the cytoplasm to the nucleus upon pathway activation. For the MAPK/ERK pathway, the *ERK2* reporter was used (Cohen‐Saidon *et al*., 2009); for the JAK/STAT pathway, the *STAT3* reporter was used (Herrmann *et al*., 2007); for the *NFkB* pathway, the *RelA* reporter was used (Harhaj and Sun, 1999); and for the PI3K/AKT pathway, the *FOXO1* reporter was used (Van Der Heide *et al*., 2004).

#### 2.4.1 Oncogenic activity prediction

Oncogenic activity of specified VUS was measured using fluorescently labeled signaling pathway reporter for the NFkB pathway (RelA), PI3K/AKT pathway (FOXO1), JAK/STAT pathway (STAT3), or MAPK/ERK pathway (ERK2). Activation is represented by the median nuclear‐to‐cytoplasmic ratio (NCR) for each condition, and the patient variant is compared to the wild‐type form of the gene or a known oncogenic mutation. NCR values were measured using a fluorescent microscope and an accompanying image analysis algorithm. NCR is a ratio of the measured fluorescent reporter in the nucleus (active state) versus the cytoplasm. All experiments were repeated at least three times.

**Figure 1:**
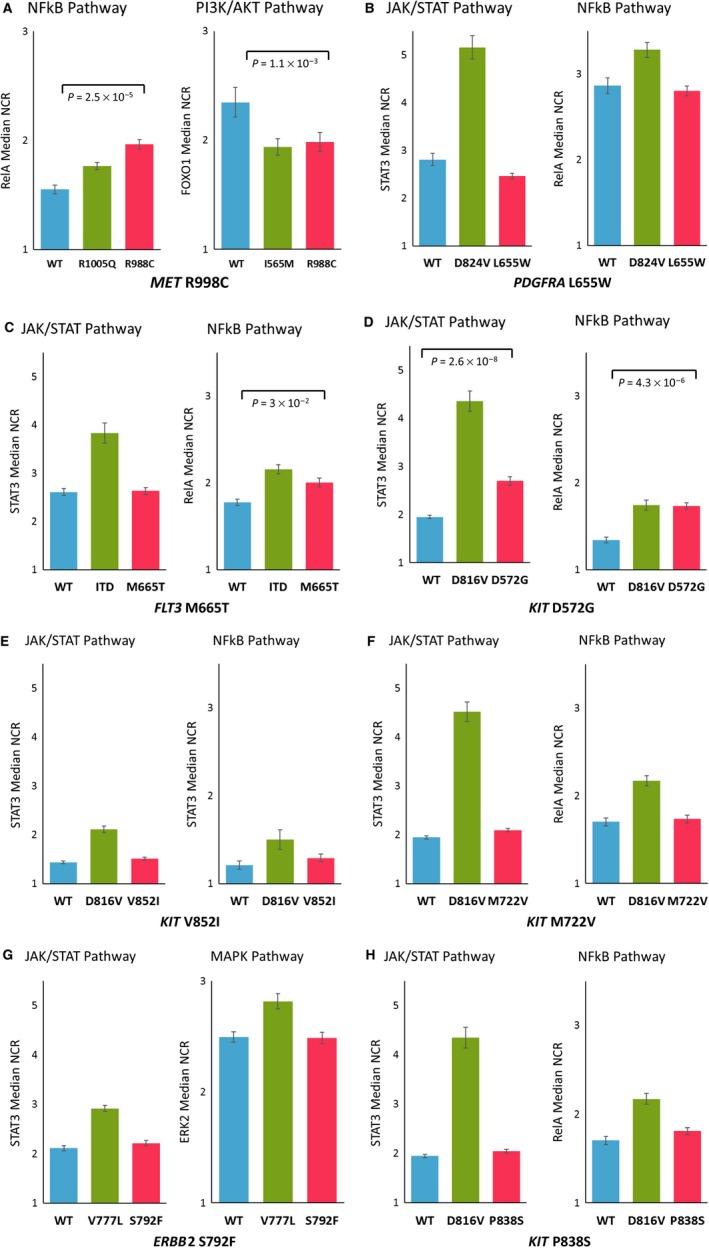
Oncogenic activities of eight variants with unknown significance (VUS) identified in patients from the SHIVA01 trial. Oncogenic activity of VUS was measured using fluorescently labeled signaling pathway reporter for the NFkB pathway (RelA), PI3K/AKT pathway (FOXO1), JAK/STAT pathway (STAT3), or MAPK/ERK pathway (ERK2). The oncogenic activities, represented by the median nuclear‐to‐cytoplasmic ratio (NCR), of patients' variants (red bars) were compared to the oncogenic activities of wild‐type (blue bar) or known mutations (green bar). Patient VUS were considered active when a 20% variation of the NCR compared to the wild‐type was observed. Significance is calculated using a Student's *t*‐test.

NCR values were normalized and scored according to the activation levels of WT and known mutations, so that 0% represents WT activity and 100% is the activity of a known mutation. This was achieved using standard rescaling methods: score = (VUS – WT)/(MT‐WT), where VUS is the reported NCR of the VUS condition, WT is the reported NCR of the wild‐type condition, and MT is the reported NCR of the known mutation condition. Only VUS that had an oncogenic activity ≥20% were considered to be active. Statistical significance between the VUS activity and the WT activity was calculated using a Student's *t*‐test (Fig. 1) (Hong *et al*., 2016) (Golbstein *et al*., 2017).

#### 2.4.2 MTA response prediction

**Figure 2:**
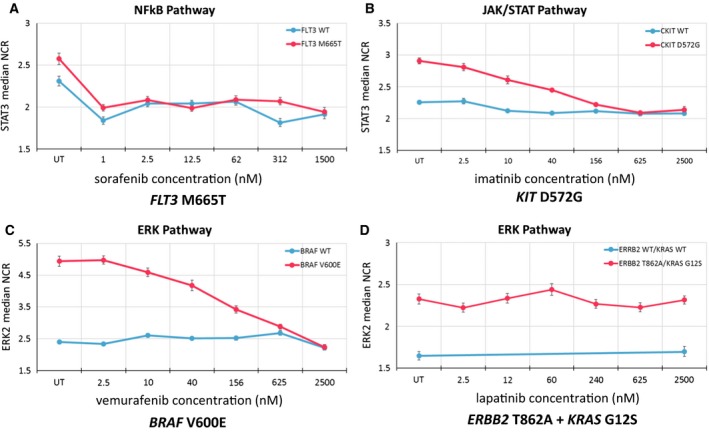
Examples of *in vitro* molecularly targeted therapy dose–response profiles of active variants identified in patients from the SHIVA01 trial. In panels A–C, sorafenib, imatinib, and vemurafenib were able to inhibit the oncogenic activities of *FLT3* M665T, *KIT* D572G, and *BRAF* V600E, respectively. In panel D, lapatinib was not able to inhibit the oncogenic activity of *ERBB2* T862A in the presence of *KRAS* G12S mutation.

To measure the response of variants to the different MTAs, a 6‐point dose–response curve of the MTA was tested (sorafenib 1 nm–1500 nm, imatinib 2.5 nm–2500 nm, vemurafenib 2.5 nm–2500 nm, lapatinib 2.5 nm–2500 nm). Drugs were incubated 6 h after transfection in the corresponding doses. All experiments were repeated at least three times. Inhibition of MTA response was the same as the mutation activation cutoff described above, with the criteria of the variant oncogenic activity decreasing below 20% in any dose compared to its oncogenic activity in the untreated condition (Fig. 2).

#### 2.4.3 Survival prediction

The prediction is based on the MTA response in the FACT assay: (a) The prediction according to FACT was considered to be positive when the specified MTA in SHIVA01 inhibited the pathway found to be activated by the mutation or VUS, while (b) it was considered to be negative when the MTA failed to inhibit the signaling pathway, or the VUS was not found to be activating (Table 1).

**Table 1:** Oncogenic activity of variants, survival prediction, and PFS. MTA, molecularly targeted agent; FACT, functional annotation for cancer treatment; PFS, progression‐free survival; NR, not relevant MTA according to FACT or MTA given in the SHIVA01 trial based on another alteration

Patient	Cancer type	MTA	Variant description	Variant Type	Oncogenic activity according to FACT prediction	Survival prediction	PFS
1	Head & Neck cancer	Erlotinib^a^	MET R988C	VUS	Yes	Negative	3.7
2	Head & Neck cancer	Sorafenib	PIK3CA E545K	Known	Yes	Negative	1.3
			PDGFRA L655W	VUS	No		
3	Lung cancer	Sorafenib	FLT3 M665T	VUS	Yes	Positive	5.9
4	Lung cancer	Imatinib	KIT D572G	VUS	Yes	Positive	8.4
5	Colorectal cancer	Vemurafenib	BRAF V600E	Known	Yes	Positive	5.6
6	Ovarian cancer	Imatinib	KIT V852I	VUS	No	Negative	3.7
7	Hepatocellular carcinoma	Imatinib	KIT M722V	VUS	No	Negative	1.5
8	Neuroendocrine cancer	Lapatinib + trastuzumab	ERBB2 T862A	Known	Yes	Negative	1.1
			KRAS G12S	Known	Yes		
9	Lung cancer	Lapatinib + trastuzumab^b^	EGFR E746_T751 > A	Known	Yes	Positive	2.0
10	Colorectal cancer	Lapatinib + trastuzumab	ERBB2 S792F	VUS	No	Negative	1.9
			KRAS G12D	Known	Yes		
11	Colorectal cancer	Sorafenib^c^	PIK3CA E545K	Known	Yes	Negative	2.3
12	Melanoma	Imatinib	KIT P838S	VUS	No	Negative	1.6
			NRAS Q61L	Known	Yes		

a Erlotinib based on EGFR amplification.

b Lapatinib + trastuzumab based on ERBB2 amplification.

c PDGFRA amplification.

### 2.5 Statistical analyses

PFS was defined as the time from the beginning of the MTA to the first documentation of disease progression on treatment or deaths. Because all patients underwent a progression, a nonparametric Kruskal–Wallis test was used to assess differences between positive and negative prediction groups. PFS of the two groups was represented using the method of Kaplan–Meier.

## 3 Results

The variant profiles of 12 patients (Table 1) treated with MTAs in the RTK/MAPK pathway in the SHIVA01 trial were selected to assess oncogenic activities of VUS and identified mutations (well‐known pathogen variants) as positive controls via microscopic quantification of nuclear signaling protein localization (Golbstein *et al*., 2017; Hong *et al*., 2016) using NovellusDx Functional Annotation for Cancer Treatment (FACT). FACT allows measuring the *in vitro* oncogenic activity of mutations and VUS, alone or in combination, in the presence or absence of the MTAs administered.

FACT uncovered the oncogenic activity of eight VUS (Fig. 1) and eight previously annotated mutations in the 12 patients in the RTK/MAPK group, as well as combinations of mutations and VUS within this group. We showed that VUS in *PDGFRA* (L655W), *ERBB2* (S792F), and three of the four *KIT* VUS were not activating variants when looking to their respective downstream signaling pathways. On the other hand, the VUS in *MET* (R988C), *FLT3* (M665T), and *KIT* (D572G) were found to be activating (Table 1). The eight well‐known mutations were all categorized as activating (data not shown).

Following assessment of the oncogenic activity of variants, MTA response prediction was measured *in vitro*, blinded to the actual clinical trial results. MTA response was not tested according to FACT in patients where (a) MTA given in SHIVA01 was based on another alteration (patients 1, 9, and 11) and (b) the VUS did not show an oncogenic activity (patients 2, 6, 7, 10, and 12). For the latter patients, survival prediction was considered negative except for patient 9 who was treated with lapatinib + trastuzumab based on *ERBB2* amplification where the prediction was considered positive in accordance with the literature. MTA response was then assessed according to FACT in four patients; the MTA efficiently inhibited the pathway in three patients (3, 4, and 5) where survival prediction was positive, but not in one patient (8) where survival prediction was negative (Fig. 2 and Table 1).

**Figure 3:**
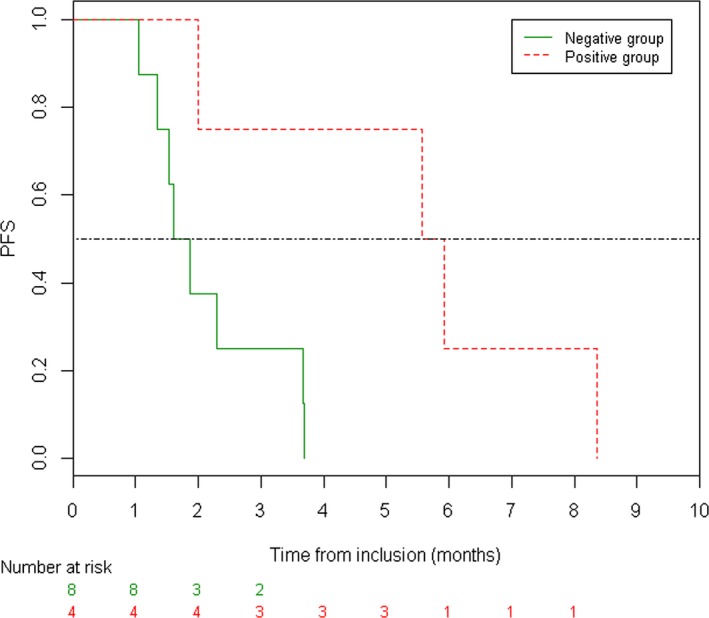
Progression‐free survival in negative and positive prediction groups according to the functional assay.

Four of 12 patients (33%) had a positive prediction, while the remaining eight patients (66%) had a negative prediction that may be explained by the presence of a potential resistance alteration to tyrosine kinase receptor inhibitors such as *PIK3CA* mutation (patients 2 and 11), *KRAS* mutations (patients 8, 10, and 12), inappropriate MTA used (patient 1), or VUS without oncogenic activity (patients 6 and 7). Survival predictions according to FACT were then correlated with the actual PFS of the SHIVA01 patients (Fig. 3). Positive patients had a median PFS of 5.8 months versus 1.7 months in negative patients (*P* < 0.05 using a Kruskal–Wallis nonparametric test to compare the PFS between the two groups).

## 4 Discussion

Our results highlight the role of functional interpretation of molecular profiles, enabling more accurate prediction of response to MTAs. This study also exemplifies the predictive power of an innovative *in vitro* functional assay to assess the oncogenic activity of mutations or VUS and more importantly to predict the response of MTAs in the presence of these mutations or VUS and variant combination. The complex interactions of genetic alterations in tumors represent a major challenge for precision medicine; complicating treatment algorithm designs and the selection of driver events to be targeted by MTAs (Le Tourneau *et al*., 2016). It is therefore crucial to develop *in vitro* or *in vivo* systems that allow testing treatment hypotheses before administration to patients. The mouse avatar concept (Malaney *et al*., 2014), and 3D culture methods (Pauli *et al*., 2017), might be used to assess the safety and efficacy profiles of MTAs or MTA combinations, albeit with significant limitations. FACT constitutes an alternative approach to the implementation of *in vitro* assays to help interpreting molecular alterations and assess MTA response to guide treatment. Our results suggest that the hypothesis driving the SHIVA01 trial might be positive by the addition of the functional interpretation of the variants. Similar assays are reported in the literature enabling the measurements of variants' oncogenic activities and the efficacy of MTA on inhibiting signaling pathways. The mixed‐all‐nominated‐mutants‐in‐one (MANO) method recently reported allows evaluating *in vitro* the oncogenic activity and drug sensitivity of VUS in a high‐throughput manner (Kohsaka *et al*., 2017). The application of this new method on 101 nonsynonymous *EGFR* variants allowed the discovery of a number of new mutations conferring resistance to *EGFR* tyrosine kinase inhibitors and pinpointed *EGFR* mutations that should rather be targeted by cetuximab. The authors lacked however clinical data to validate the prediction of the MANO method. Naturally, this method will require further validation prospectively in a cotrial design to validate its prospective feasibility in time frames compatible with clinical practice. Ideally *in vitro* functional assays need to be adapted to any type of molecular alteration including gene amplifications (known to be driver alterations such as *ERBB2* in breast cancer), translocations (such as *ALK* in lung cancer), and deletions of tumor suppressor genes (such as *PTEN*).

## Conflict of interest

GC, OE, ZB, BM, and MV are full‐time employees of NovellusDx.
